# Diabetic Myonecrosis: Uncommon Complications in Common Diseases

**DOI:** 10.1155/2014/175029

**Published:** 2014-03-10

**Authors:** Sisira Sran, Manpreet Sran, Nicole Ferguson, Prachi Anand

**Affiliations:** ^1^Department of Medicine, Nassau University Medical Center, 2201 Hempstead Turnpike, East Meadow, NY 11554, USA; ^2^New York Institute of Technology, College of Osteopathic Medicine, Northern Boulevard, P.O. Box 8000, Old Westbury, NY 11568-8000, USA; ^3^New York Institute of Technology, College of Osteopathic Medicine, Nassau University Medical Center, East Meadow, NY 11554, USA; ^4^Department of Rheumatology, Nassau University Medical Center, 2201 Hempstead Turnpike, East Meadow, NY 11554, USA

## Abstract

We report a case of sudden thigh pain from spontaneous quadriceps necrosis, also known as diabetic myonecrosis, in a 28-year-old patient with poorly controlled diabetes mellitus. Diabetic muscle infarction is a rare end-organ complication seen in patients with poor glycemic control and advanced chronic microvascular complications. Proposed mechanisms involve atherosclerotic microvascular occlusion, ischemia-reperfusion related injury, vasculitis with microthrombi formation, and an acquired antiphospholipid syndrome. Diabetic myonecrosis most commonly presents as sudden thigh pain with swelling and should be considered in any patient who has poorly controlled diabetes mellitus.

## 1. Background

Diabetic myonecrosis is a rare complication associated with poorly controlled diabetes and advanced microvascular disease. Diabetic muscle infarction is usually unilateral and affects the lower limbs. The most commonly affected muscles are quadriceps, hip adductors, and hamstrings [1]. Bilateral involvement has been reported in 8.4% cases [2]. There has been one case reported of bilateral upper limb involvement [3]. It presents clinically as sudden pain and swelling of the involved muscle without previous trauma or fever. The pain is usually present at rest. Although diabetes may be a common disease, diabetic myonecrosis is a rare complication. We report the presentation of diabetic myositis in a 28-year-old male patient.

## 2. Case Report

A 28-year-old African American male with uncontrolled type II diabetes mellitus arrived at the emergency department with sudden pain in the right lower extremity and difficulty bearing weight. The patient described the pain as aching, 10 out of 10 during maximal intensity with no relation to movement and no alleviating factors. He denied any preceding trauma or similar episodes in the past. On admission, he was afebrile with a pulse rate of 124 and blood pressure of 113/78. Examination of the left inner thigh revealed erythema, warmth, and a nonhealing, circumferential wound with no evidence of discharge or induration. The right lower extremity was unremarkable. Laboratory values revealed an elevated leukocyte count of 14.82 cells/mcl, with neutrophils of 84%. Random glucose was 660 mg/dL, K^+^ 3.7 mmol/liter, and Na^+^ 129 mmol/liter with an anion gap of 20. Other laboratory findings included a serum lactate level of 4.7 with creatine kinase levels of 176/U/L. Hemoglobin A1c was 16%. Antinuclear antibody (Ab), jo-1 Ab, scl-70 Ab, proteinase 3a-Ab, and myeloperoxidase Ab were negative. Serology was negative for HIV and hepatitis A, B, and C and there was no bacterial or fungal growth on blood cultures.

Computerized tomography of the left thigh revealed irregular internal contrast enhancement and hypoattenuation of the vastus medialis. A complex suprapatellar joint effusion was also noted. Magnetic resonance imaging of the same area revealed generalized subcutaneous edema with diffuse signal abnormality involving the vastus medialis, lateralis, and intermedius muscles suggesting diffuse inflammatory changes. An ultrasound guided biopsy of the left thigh muscle was performed. Pathology showed necrotic and atrophic skeletal muscle infiltrated by necrotic neutrophils. A fragment of extensively fibrotic and atrophic skeletal muscle with mild mononuclear inflammation and hemosiderin deposition was noted. Gram stain was negative for bacteria. The patient received adequate pain control throughout the hospital course with management aimed towards achieving optimal glycemic control.

## 3. Discussion

The pathophysiology of diabetic myonecrosis is not well understood but it has been attributed to thromboembolic events secondary to microvascular endothelial damage leading to tissue ischemia, which then triggers an inflammatory cascade leading to local tissue damage and ischemic necrosis. Reperfusion of ischemic tissues associated with endothelial dysfunction is manifested as impaired endothelium-dependent dilation in arterioles along with increased oxygen radicals, with less nitric oxide, following reperfusion [4]. The resulting imbalance between superoxide and nitric oxide in endothelial cells leads to the production and release of inflammatory mediators (tumour necrosis factor and platelet-activating factor) along with increased biosynthesis of adhesion molecules [4]. The inflammatory cascade increases intracompartmental ischemia from edema with subsequent tissue necrosis. An acquired antiphospholipid syndrome resulting in disruption of clotting and pathways superimposed on endothelial dysfunction may also be a contributory factor in the progression of microvascular disease, acting as a link between immunological and haemostatic system in the pathogenesis of diabetic myonecrosis [1].

In the workup of diabetic myonecrosis routine laboratory investigations are relatively nonspecific. There may be leukocytosis and serum creatine kinase levels may remain normal or slightly elevated. MRI is the most sensitive diagnostic modality, and in the appropriate clinical setting muscle biopsy is not required. The characteristic feature is an increased signal from the affected muscle area (intramuscular and perimuscular tissues) in T2-weighted, inversion-recovery, and gadolinium-enhanced images and isointense or hypointense areas on T1-weighted images, secondary to increased water content from edema and inflammatory changes that accompany the infarction [5]. The most accurate diagnostic modality is by tissue biopsy. On biopsy it grossly appears as nonhemorrhagic pale muscle tissue. Histologically, there are large areas of muscle necrosis and edema, phagocytosis of necrotic muscle fibres, granular tissue, and collagen [6]. This is followed by eventual replacement of necrotic muscle fibers by fibrous tissue, myofiber regeneration, and mononuclear cell infiltration [6].

Treatment is conservative with supportive measures aimed at pain control with analgesics along with maintaining target glycemic control. Non-weight-bearing activity and physical rehabilitation may be useful after the acute phase. Those who underwent surgery had an average recovery period of 13 weeks compared to 5.5 weeks for those only received conservative treatment [7]. Once a patient with diabetes develops diabetic myonecrosis, the likelihood of recurrence in the same muscle or contralateral limb is greater than 50%, with as many as 1 to 2 episodes per year after the initial event [7]. Although diabetic myonecrosis has a good prognosis, it is an indicator of poor long-term prognosis. A recent review found that most patients die within 5 years of diagnosis as diabetic muscle infarction suggests substantial vascular compromise [7].

## 4. Conclusion

Although diabetes may be a common disease, diabetic myonecrosis is a rare complication and indicator of poor long-term prognosis in diabetic patients with poor glycemic control. Presentation of diabetic myonecrosis has an average age of 40; our patient illustrates that myonecrosis can occur at much younger ages and reenforces the need for frequent follow-up with diabetic patients and achievement of appropriate glycemic control. Diabetic myonecrosis should be included on the differential diagnosis for diabetic patients of any age presenting with extremity pain and swelling.
